# Seasonal environmental variation and its association with virulence and antimicrobial resistance in aquatic *Pseudomonas aeruginosa*

**DOI:** 10.1186/s12917-026-05532-6

**Published:** 2026-05-13

**Authors:** Aya El Badawy, Dalia Hamza, Zeinab Ahmed, Maha A. Sabry

**Affiliations:** Department of Zoonoses, Faculty of Veterinary Medicine, Cairo University, PO Box 12211, Giza, Egypt

**Keywords:** Seasonal variation, Aquaculture, *P. aeruginosa*, Virulence genes, Antimicrobial resistance genes, Egypt

## Abstract

Aquaculture is vital for global food security, but climate change threatens fish health by altering environmental conditions. *Pseudomonas aeruginosa* is a pathogenic bacterium causing significant losses in fish farms. This study investigated the effects of seasonal water temperature and pH variations on its prevalence, virulence, and antibiotic resistance in Nile Tilapia. A total of 328 Nile tilapia samples from two Egyptian farms were pooled into 82 composite samples. *P. aeruginosa* was isolated on selective agar, identified through biochemical tests, and confirmed by PCR targeting the *gyrB* gene. Virulence genes (*opr*L, *exo*S, *phz*M, *tox*A) and resistance genes (ESBL: *bla*_TEM_, *bla*_SHV,_ *bla*_CTX−M_, *bla*_OXA−1_; carbapenemases: *bla*_KPC_, *bla*_NDM_, *bla*_VIM_, *bla*_OXA−48_) were detected, and correlations with water temperature and pH were analyzed. Phylogenetic analysis of *opr*L sequences assessed genetic relatedness to human strains. *Pseudomonas aeruginosa* was detected in the vast majority of samples, with prevalence remaining high throughout the year, peaking numerically in summer. The prevalence of virulence genes in *P. aeruginosa * varied across seasons. *exo*S was consistently detected in all seasons, whereas *tox*A and *opr*L were most prominent in summer. *phz*M showed marked seasonal variation, with the highest occurrence in summer. Antibiotic resistance genes were most prominent in the warmer months, with *bla*_TEM_ consistently present, *bla*_CTX−M_ highly prevalent, and *bla*_OXA−48_ showing a noticeable increase. Phylogenetic analysis revealed genetic similarity between fish-derived isolates and previously reported human strains, suggesting potential zoonotic relevance. These findings highlight the widespread occurrence of *P. aeruginosa * and the seasonal distribution of virulence and antimicrobial resistance genes in aquaculture systems. Continuous monitoring and improved management strategies are recommended to limit the spread of resistant strains.

## Introduction

Aquaculture is increasingly critical for global food security, contributing about one-third of the world’s fish supply and helping meet rising protein demands [1, 2]. However, climate change threatens aquaculture sustainability by altering environmental conditions, from cellular functions to ecosystems, impacting fish health and increasing disease risk [3–6]. Rising temperatures and declining pH, driven by ocean acidification, create stressful conditions that disrupt fish physiology, reduce resilience, and facilitate the emergence and spread of antibiotic-resistant pathogens [7–10].

Among these pathogens, *Pseudomonas aeruginosa* poses a major threat to freshwater and marine aquaculture, causing diseases such as ulcerative syndrome and septicemia, with mortality rates up to 50% and economic losses due to poor growth and flesh quality [11, 12]. Beyond fish, *P. aeruginosa* is an opportunistic human pathogen, causing respiratory, urinary tract, wound, and nosocomial infections, particularly in immunocompromised individuals [13]. The bacterium thrives at temperatures up to 42 °C and responds to environmental changes by altering gene expression, metabolism, motility, and membrane structure, increasing its zoonotic potential through contaminated water or fish products [14–18].

*Pseudomonas aeruginosa* possesses a diverse set of virulence factors that facilitate host invasion, immune evasion, and survival in both clinical and environmental niches [18]. Exotoxin A, encoded by the *tox*A gene, disrupts host protein synthesis by ADP-ribosylating elongation factor 2 (EF-2), resulting in cellular damage and death [19]. The *exo*S gene encodes Exoenzyme S, a type III secretion system effector that interferes with the cytoskeleton and cell signaling via GTPase-activating and ADP-ribosyltransferase activities, triggering apoptosis and impairing immune responses [20]. The *phz*M gene contributes to the synthesis of pyocyanin, a redox-active pigment that produces reactive oxygen species (ROS), leading to oxidative stress, inflammation, and disruption of cellular respiration [21, 22]. The *opr*L gene encodes an outer membrane lipoprotein that is critical for maintaining membrane integrity, biofilm formation, and resistance to both antibiotics and environmental stressors [23]. Additional virulence determinants include elastases LasA and LasB, which degrade extracellular matrix components to facilitate tissue invasion, and alginate production, which strengthens the biofilm matrix and protects against host defenses and antimicrobial agents [24]. These virulence genes are frequently upregulated under environmental stressors, such as elevated temperatures, enhancing the pathogen’s fitness in aquaculture environments and increasing its zoonotic potential [25].

*Pseudomonas aeruginosa* is well-known for its resistance to a broad range of antibiotics, posing a significant public health threat [26]. The bacterium can transmit multidrug-resistant (MDR) plasmids to humans via consumption of contaminated or undercooked fish and fish products [27]. The widespread overuse and misuse of antibiotics in both human medicine and animal husbandry, including aquaculture, has disrupted microbial ecosystems and driven the emergence of antimicrobial resistance (AMR), now recognized as a global health crisis [28].

A key mechanism underlying AMR in *P. aeruginosa* is the acquisition and expression of potent β-lactamase genes, which hydrolyze the β-lactam ring of antibiotics, preventing their binding to penicillin-binding proteins (PBPs) and rendering these drugs ineffective [29]. Among these, extended-spectrum β-lactamases (ESBLs) such as *bla*_TEM_, *bla*_CTX−M_, *bla*_SHV_, and *bla*_OXA−1_ confer resistance to a wide range of β-lactam antibiotics. While ESBLs are typically associated with *Enterobacteriaceae*, they are increasingly identified in *P. aeruginosa* isolates [29, 30]. ESBL production can facilitate the development of carbapenem resistance, especially when combined with mechanisms like reduced outer membrane permeability or efflux pump overexpression [30]. Resistance to carbapenems considered last-resort antibiotics for multidrug-resistant Gram-negative infections is rising and is mainly mediated by carbapenemases, including *bla*_KPC_, *bla*_OXA−48_, *bla*_VIM_, and *bla*_NDM_, which hydrolyze these antibiotics and contribute to global dissemination of resistance [31–33].

Environmental stressors, particularly elevated temperatures, further exacerbate AMR by accelerating bacterial growth, triggering heat-shock responses, and promoting horizontal gene transfer, thereby enhancing the risk of resistance transmission [34, 35]. These findings highlight the urgent need for alternative strategies and robust antimicrobial stewardship programs in aquaculture systems [36].

Understanding the environmental dynamics of *P. aeruginosa* is critical for maintaining fish health, ensuring food safety, and limiting zoonotic AMR transmission. Despite the central role of temperature and pH in aquaculture, their specific effects on the prevalence, virulence, and resistance of *P. aeruginosa* in Egyptian fish farms remain poorly characterized. Accordingly, this study investigates how seasonal variations in temperature and pH influence the prevalence, antimicrobial resistance patterns, and virulence gene profiles (*opr*L, *exo*S, *phz*M, *tox*A) of *P. aeruginosa* isolated from intensively farmed Nile tilapia, while also examining the potential zoonotic implications of these isolates.

## Materials and methods

### Ethical statement

This study was conducted following the ethical guidelines of the Faculty of Veterinary Medicine, Cairo University, Egypt, and received approval from the Institutional Animal Care and Use Committee (IACUC), approval number: VET-CU-IACUC-131,020,241,011.

### Sample collection and processing

A total of 328 Nile tilapia (*Oreochromis niloticus*) were randomly collected from two Egyptian fish farms with similar environmental conditions across five seasonal sampling periods: winter, spring, early summer, summer, and autumn. Water temperature and pH were measured during each sampling period at multiple time points to capture seasonal variability, using standardized, calibrated equipment. Measurements were performed in the morning between 8:00 and 10:00 AM. Temperature was recorded using liquid crystal display (LCD) aquarium thermometers, and pH was measured with a Fisher Scientific pH meter (Waltham, MA, USA), following manufacturer instructions. To improve spatial representativeness, readings were taken from multiple locations within each pond, and seasonal values represent the average of all measurements.

Based on climatic data, samples were categorized into five groups: Group 1 (Winter: 22 °C, pH 7.5, February), Group 2 (Spring: 28 °C, pH 8.0, March), Group 3 (Early Summer: 35 °C, pH 6.5, May), Group 4 (Summer: 39 °C, pH 5.5, July), and Group 5 (Autumn: 37 °C, pH 6.0, September). Fish were pooled into 82 groups of four individuals each, distributed as follows: 15 in winter, 18 in spring, 18 in early summer, 16 in summer, and 15 in autumn. All analyses were performed using the pooled samples (*N* = 82) as the analytical unit, and prevalence was expressed as the proportion of positive pooled samples within each season. Samples were transported in sterile, refrigerated containers to preserve integrity and prevent contamination.

Each pooled group was subjected to examination of the skin, intestinal tract, and muscle tissues. Skin samples were obtained using sterile cotton swabs, while intestinal and muscle tissues were aseptically excised following surface sterilization with a red-hot scalpel. Samples from each group were then combined into a single pooled specimen for subsequent isolation and identification of *Pseudomonas* spp.

In this study, tilapia was euthanized by rapid cooling in water below 4 °C to reduce distress. After euthanasia, the specimens were disposed of by incineration.

### Isolation and identification of suspected *Pseudomonas* spp.

Pooled samples were enriched in fresh tryptic soy broth (HiMedia Laboratories, Mumbai, India) at 37 °C for 18–24 h. Enriched cultures were streaked onto Pseudomonas base agar supplemented with 5 mL glycerol and CN supplement (Cetrimide and Nalidixic Acid; Biolife Italiana, Milan, Italy) and incubated aerobically at 37 °C for 24 h [37]. Suspected colonies were subcultured on the same medium to obtain pure isolates, which were examined for colony morphology.

For biochemical identification, isolates were subjected to standard conventional tests using reagents from Oxoid Ltd. (Basingstoke, Hampshire, UK), including oxidase and catalase tests, triple sugar iron (TSI) agar, citrate utilization, urease activity, indole production, motility, oxidative/fermentative glucose test, gelatin liquefaction, and nitrate reduction [38]. These tests confirmed species-level identification and differentiated *P. aeruginosa* from other non-fermenting Gram-negative bacilli.

### Genomic DNA extraction

Genomic DNA was extracted from confirmed *Pseudomonas* isolates using the boiling method described by Oliveira et al. [39]. DNA concentration and purity were measured with a NanoDrop Spectrophotometer (Thermo Scientific, USA) and stored at − 20 °C until further analysis.

### Molecular identification of *P. aeruginosa*

All isolates were confirmed as *P. aeruginosa* by PCR amplification of the *gyrB* gene using species-specific primers [40] (Table 1). Positive controls included a reference *P. aeruginosa* strain (ATCC 27853), and negative controls consisted of no-template reactions.

**Table 1:** List of oligonucleotide sequences and their PCR conditions of *Pseudomonas* spp. identification gene and some of its virulence genes

Target genes (bp)	Primer sequences (5ʹ–3ʹ)	Cycling Conditions	References
***gyrB*** **gene** (367 bp)	**F**:AAGTACGAAGGCGGTCTGAA **R**:GTTGTTGGTGAAGCAGAGCA	95 °C for 2 min, 30 cycles of 92 °C for 60 s, 55 °C as annealing temperature and 72 °C for 1 min, and finally One cycle at 72 °C for 8 min	[40]
***opr*L** (504 bp)	**F**: ATG GAA ATG CTG AAA TTC GGC **R**:CTT CTT CAG CTC GAC GCG ACG	94˚C for 5 min; 40 cycle (96 ˚C for 1 min, 55 ˚C for 1 min ,72 ˚C for 1 min), 72 °C for 10 min.	[21]
***tox*A** (396 bp)	**F**: GAC AAC GCC CTC AGC ATC ACC **R**: AGC CGC TGG CCC ATT CGC TCC AGC GCT	94˚C for 5 min; 30 cycle (94 ˚C for 1 min, 55 ˚C for 1 min ,72 ˚C for 1 min), 72 °C for 10 min.	
***exo*S** (118 bp)	**F**: GCG AGG TCA GCA GAG TAT CG TTC **R**: GGC GTC ACT GTG GAT GC	94˚C for 5 min; 36 cycle (94 ˚C for 30 s, 58 ˚C for 30 s ,68 ˚C for 1 min), 72 °C for 10 min.	
***phz*M** (875 bp)	**F**: ATG GAG AGC GGG ATC GAC AG ATG **R**: CGG GTT TCC ATC GGC AG	94˚C for 5 min; 30 cycle (94 ˚C for 30 s, 54 ˚C for 30 s ,72 ˚C for 1 min), 72 °C for 10 min.	

### Molecular detection of virulence genes in *P. aeruginosa*

Virulence gene detection was performed in all confirmed *P. aeruginosa* isolates using uniplex PCR with four sets of primers targeting the *opr*L, *exo*S, *phz*M, and *tox*A genes. Each 25 µL PCR reaction contained 3 µL of template DNA, 12.5 µL of Emerald Amp MAX PCR master mix (Takara, Japan), 0.5 µL of each primer (10 pmol/µL; Metabion, Germany), and PCR-grade water to complete the final volume.

PCR products were electrophoresed on a 1.5% agarose gel at 100 volts for 45 min. DNA bands were visualized under UV transillumination (UVP GelDoc-It Imaging System, Analytik Jena, USA) after staining with ethidium bromide (Sigma-Aldrich, USA). The amplification conditions and specific primer sequences are presented in Table 1.

### Molecular detection of β-Lactamase-Encoding Genes (ESBLs)

Detection of *bla*_TEM_, *bla*_SHV_, *bla*_CTX−M_, and *bla*_OXA−1_ genes was performed by multiplex polymerase chain reaction (PCR) on all *P. aeruginosa* isolates using primers listed in Table 2. Each reaction was prepared in a total volume of 25 µL, consisting of 3 µL of template DNA, 12.5 µL of Emerald Amp MAX PCR Master Mix (Takara, Japan), 0.5 µL of each primer (10 pmol/µL; Metabion, Germany), and PCR-grade water to make up the final volume.

**Table 2:** The sequence of oligonucleotide primers used for PCR amplification of β lactamase (ESBLs) and carbapenemase encoding genes

Target genes (bp(	Primer sequences (5ʹ–3ʹ)			Cycling Conditions	Refrences
**β lactamase (ESBLs)- encoding genes**					
***bla***_**TEM**_ (445 bp)		**F**:CGCCGCATACACTATTCTCAGAATGA **R**:ACGCTCACCGGCTCCAGATTTAT	95 °C for 5 min; 30 cycles (94 °C for 30 s , 62 °C for 90 s , 72 °C for 60 s ), 72 °C for 10 min.		[41, 42]
***bla***_**SHV**_ (237 bp)		**F**:CTTTATCGGCCCTCACTCAA **R**:AGGTGCTCATCATGGGAAAG			
***bla***_**CTX−M**_ (593 bp)		**F**:ATGTGCAGYACCAGTAARGTKATGGC **R**:TGGGTRAARTARGTSACCAGAAYC AGC GG			
***bla***_**OXA−1**_ (813 bp		**F**:ACA CAA TAC ATA TCA ACT TCG C **R**:AGT GTG TTT AGA ATG GTG ATC			
**Carbapenemase- encoding genes**					
***bla***_**KPC**_ (882 bp)		**F**:ATG TCA CTG TAT CGC CGT CT **R**: TTT TCA GAG CCT TAC TGC CC	30 cycles (94 °C for 1 min , 55 °C for 1 min, 72 °C for 2 min ), 72 °C for 10 min.		[43]
***bla***_**NDM**_ (621 bp)		**F**:GGT TTG GCG ATC TGG TTT TC **R**:CGG AAT GGC TCA TCA CGA TC			
***bla***_**VIM**_ (261 bp)		**F**:AGT GGT GAG TAT CCG ACAG **R**:ATG AAA GTG CGT GGA GAC	35 cycles ( 94 °C for 30 s , 55 °C for 30 s, 72 °C for 1 min) , 72 °C for 10 min.		[44]
***bla***_**OXA−48**_ (283 bp)		**F** :GCTTGATCGCCCTCGATT **R**: GATTTGCTCCGTGGCCGAAA	94 °C for 10 min; 30 cycles (94 °C for 40 s , 60 °C for 40 s, 72 °C for 1 min) , 72 °C for 7 min.		[45]

The PCR amplification conditions were as follows: initial denaturation at 95 °C for 5 min, followed by 30 cycles of denaturation at 94 °C for 30 s, annealing at 62 °C for 90 s, and extension at 72 °C for 60 s. A final extension step was carried out at 72 °C for 10 min [41, 42].

### Molecular detection of Carbapenemase-Encoding Genes (*bla*_KPC_, *bla*_OXA−48_, *bla*_VIM_, and *bla*_NDM_)

Multiplex PCR was used to detect the *bla*_KPC_ and *bla*_NDM_ genes in all *P. aeruginosa* isolates using specific oligonucleotide primers (Table 2). Each PCR reaction had a total volume of 25 µL and included 3 µL of template DNA, 12.5 µL of Emerald Amp MAX PCR Master Mix (Takara, Japan), 0.5 µL of each primer (10 pmol/µL; Metabion, Germany), and PCR-grade water to complete the volume.

The thermal cycling conditions for amplification were as follows: 30 cycles of denaturation at 94 °C for 1 min, annealing at 55 °C for 1 min, extension at 72 °C for 2 min, followed by a final extension at 72 °C for 10 min [43].

In contrast, *bla*_VIM_ and *bla*_OXA−48_ genes were detected using uniplex PCR with specific oligonucleotide primers (Table 2). Amplification of the *bla*_VIM_ gene followed the protocol described by Li et al. [44], involving 35 cycles of 94 °C for 30 s, 55 °C for 30 s, and 72 °C for 1 min, with a final extension at 72 °C for 10 min.

For the *bla*_OXA−48_ gene, the thermal cycling conditions were adapted from Dallenne et al. [45] and included an initial denaturation at 94 °C for 10 min, followed by 30 cycles of 94 °C for 40 s, 60 °C for 40 s, and 72 °C for 1 min, with a final extension at 72 °C for 7 min.

### Molecular characterization and phylogenetic analysis of the *opr*L Gene in *P. aeruginosa*

The amplified fragments of two randomly selected *P. aeruginosa * isolates were purified using the QIAquick Gel Extraction Kit (QIAGEN, Germany) according to the manufacturer’s instructions. Sequencing was conducted using forward and reverse primers specific to the *opr*L gene. The obtained sequences were submitted to the National Center for Biotechnology Information (NCBI) GenBank under accession numbers PQ855211 and PQ855212.

To explore genetic similarity, the sequenced *opr*L genes from fish-derived *P. aeruginosa* isolates were compared with publicly available sequences from human clinical strains, including those from critical care patients, using the NCBI BLAST tool. Relevant sequences were retrieved from GenBank, aligned using CLUSTALW in BioEdit version 7.0.1.4, and subjected to phylogenetic analysis in MEGA X. A bootstrap consensus tree was constructed from 1000 replicates to ensure robustness and reliability.

### Statistical analysis

For statistical analysis, data from the 82 pooled samples were used to assess associations between sampling month and *P. aeruginosa* prevalence. Cramer’s V was calculated, with interpretation thresholds as follows: values ≤ 0.2 were considered to indicate a weak association, > 0.2 to ≤ 0.6 a moderate association, and > 0.6 a strong association. In addition, the Pearson Chi-square test was applied to assess the statistical significance of the association between variables. The prevalence of *P. aeruginosa* across different sampling months was expressed with 95% confidence intervals. All statistical analyses were conducted using PASW Statistics software, version 18.0 (SPSS Inc., Chicago, IL, USA), with the level of significance set at *p* < 0.05. Pearson’s correlation coefficient (*r*) and regression analysis (*R²*) tests were performed to evaluate the association between the studied variables.

## Results

### Occurrence of *P. aeruginosa*

**Fig. 1:**
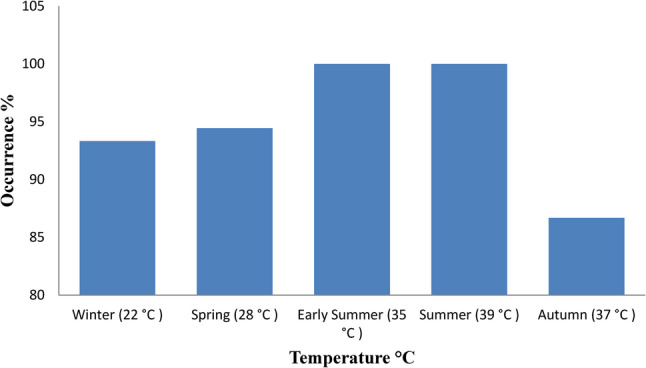
Occurrence of *P. aeruginosa* in relation to fishpond water temperature. The bar chart shows prevalence of *P. aeruginosa* across seasonal groups with corresponding pond water temperatures

**Fig. 2:**
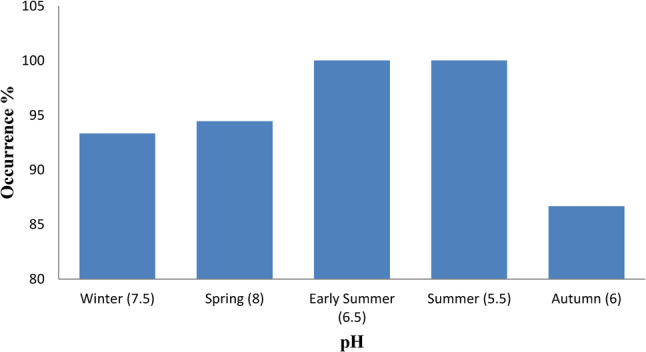
Occurrence of *P. aeruginosa* in relation to pond water pH. The bar chart shows prevalence of *P. aeruginosa* across seasonal groups with corresponding pH values

Out of 82 pooled fish samples from two Egyptian farms, 78 were confirmed as *P. aeruginosa*, with an overall prevalence of 95.12% (95% CI: 87.74–98.46). Seasonal prevalence showed a moderate association (Cramer’s V = 0.23, *p* = 0.383), slightly lower in winter (93.33%) and spring (94.44%), peaking at 100% in early summer and summer, and declining to 86.67% in autumn (Table 3). Pearson correlation revealed weak positive and negative associations with pond temperature (*r* = 0.18, *p* = 0.778) and pH (*r* = − 0.14, *p* = 0.821), respectively. Detection tended to be higher at elevated temperatures (35 and 39 °C) and lower pH levels (5.5 and 6.5), but these trends were not statistically significant, with the lowest detection observed at 37 °C and pH 6 in autumn (Figs. 1 and 2).

**Table 3:** Seasonal distribution and occurrence of *P. aeruginosa* in fish from different environmental groups

Different environmental groups	Sampling periods	No. of pooled fish	Positive for *P*. aeruginosa		(95%CI)
			No	%	
Group 1	Winter	15	14	93.33	(68.16–99.99)
Group 2	Spring	18	17	94.44	(72.35–99.99)
Group 3	Early Summer	18	18	100	(79.33–100)
Group 4	Summer	16	16	100	(77.31–100)
Group 5	Autumn	15	13	86.67	(60.86–97.52)
Total	-	82	78	95.12	(87.74–98.46)

### Distribution of virulence Genes, β-Lactamase, and Carbapenemase -Encoding Genes

**Fig. 3:**
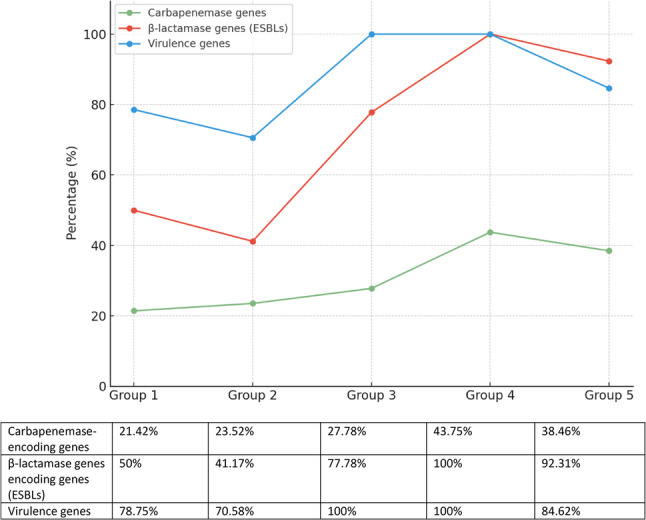
Percentage of *P. aeruginosa* isolates carrying virulence and resistance genes. The bar chart presents detection rates of virulence genes, β-lactamase–encoding genes (ESBLs), and carbapenemase–encoding genes across five environmental groups from two Egyptian fish farms

Virulence (*opr*L, *tox*A, *exo*S, *phz*M) and resistance genes (ESBLs: *bla*_TEM_, *bla*_CTX−M_, *bla*_SHV_, *bla*_OXA−1_; carbapenemases: *bla*_KPC_, *bla*_NDM_, *bla*_VIM_, *bla*_OXA−48_) showed seasonal variation (Fig. 3; Table 4).

**Table 4:** The occurrence of virulence and antibiotic resistance genes among *P. aeruginosa* isolates from two Egyptian fish farms across different environmental groups

Gene function	Gene name	Group 1	Group 2	Group 3	Group 4	Group 5
Virulence genes	*opr*L	0 (0%)	2 (11.76%)	2 (11.11%)	14 (87.5%)	0 (0%)
	*tox*A	4 (28.57%)	1 (5.88%)	3 (16.67%)	16 (100%)	1 (7.69%)
	*exo*S	10 (71.43%)	12 (70.59%)	15 (83.33%)	16 (100%)	10 (76.92%)
	*phz*M	3 (21.43%)	2 (11.76%)	5 (27.78%)	15 (93.75%)	2 (15.38%)
β lactamase (ESBLs)- encoding genes	*bla*_TEM_	7 (50%)	9 (52.94%)	13 (72.22%)	16 (100%)	11 (84.62%)
	*bla*_SHV_	0 (0%)	0 (0%)	1 (5.56%)	1 (6.25%)	2 (15.38%)
	*bla*_CTX−M_	1 (7.14%)	0 (0%)	0 (0%)	12 (75%)	0 (0%)
	*bla*_OXA−1_	0 (0%)	0 (0%)	0 (0%)	0 (0%)	0 (0%)
Carbapenemase- encoding genes	*bla*_KPC_	0 (0%)	0 (0%)	1 (5.56%)	0 (0%)	0 (0%)
	*bla*_NDM_	1 (7.14%)	2 (11.76%)	0 (0%)	0 (0%)	1 (7.69%)
	*bla*_VIM_	0 (0%)	1 (5.88%)	1 (5.56%)	3 (18.75%)	2 (15.38%)
	*bla*_OXA−48_	2 (14.29%)	2 (11.76%)	2 (11.11%)	5 (31.25%)	3 (23.08%)

The summer season showed the highest prevalence of virulence genes, β-lactamase-encoding genes, and carbapenemase encoding genes, with virulence and β-lactamase genes approaching 100% and carbapenemase genes reaching 43.75%.

Among the virulence markers, Virulence genes were most prominent in early summer and summer, with *opr*L and *phz*M reaching their highest detection in summer (87.5% and 93.75%, respectively), *tox*A peaking at 100% in summer, and *exo*S consistently high across all seasons, reaching 100% in summer.

For β-lactamase (ESBLs)-encoding genes Among ESBL-encoding genes, *bla*_TEM_ was the most prevalent, increasing from 50% in winter to 100% in summer and slightly decreasing to 84.62% in autumn; *bla*_SHV_ remained low, ranging from 0% in winter and spring to 15.38% in autumn; *bla*_CTX−M_ peaked at 75% in summer, with minimal or no detection in other seasons; while *bla*_OXA−1_ was not detected in any season.

Carbapenemase-encoding genes were generally less prevalent but peaked in summer, with *bla*_KPC_ detected only in early summer (5.56%), *bla*_NDM_ appearing in winter (7.14%), spring (11.76%), and autumn (7.69%), *bla*_VIM_ rising from 0% in winter to 18.75% in summer, and *bla*_OXA−48_ showing the highest prevalence in summer (31.25%) with lower levels in other seasons.

### Seasonal patterns of biomarker virulence and resistance genes in *P. aeruginosa* Isolates

The most complex genetic profiles of *P. aeruginosa* isolates were detected during the summer season (Group 4), coinciding with the highest temperatures and lowest pH values. These isolates commonly harbored combinations of four or more virulence genes (*opr*L, *tox*A, *exo*S, *phz*M) along with multiple resistance determinants, including *bla*_TEM_, *bla*_CTX−M_, *bla*_VIM_, and *bla*_OXA−48_, with some isolates carrying up to seven genes simultaneously.

In contrast, isolates recovered during winter (Group 1) and spring (Group 2) exhibited lower genetic diversity, typically containing only one or two virulence genes and few resistance markers. Genetic complexity began to rise in early summer (Group 3) and remained high through autumn (Group 5), though a slight reduction in virulence gene diversity was noted compared with the summer peak (Table 5).

**Table 5:** The pattern of biomarker virulence genes and resistance genes among *P. aeruginosa* isolates

Different environmental fish groups	No. of isolates	Biomarker virence genes, β-lactamase and carbapenemase-encoding genesul
Group 1	2 2 1 1 2 2 1 2 1	*exo*S *bla*_TEM_ *tox*A, *bla*_CTX−M_ *exo*S, *bla*_NDM_ *exo*S, *bla*_TEM_ *exo*S, *phz* M *phz*M, *bla*_OXA−48_ *tox*A, *exo*S, *bla*_TEM_ *tox*A, *exo*S, *bla*_TEM_, *bla*_OXA−48_
Group 2	4 1 2 2 1 1 2 1 1	*exo*S *phz*M *bla*_TEM_ *exo*S, *bla*_TEM_ *exo*S, *bla*_TEM_, *bla*_OXA−48_ *opr*L, *tox A*,* exo* S *exo*S, *bla*_TEM_, *bla*_NDM_ *opr*L, *exo*S, *bla*_TEM_ *exo*S, *phz*M, *bla*_TEM_, *bla*_OXA−48,_ *bla*_VIM_
Group 3	4 2 1 1 1 3 1 1 1 1 1	*exo*S *bla*_TEM_ *exo*S, *bla*_TEM_ *exo*S, *bla*_TEM_, *bla*_KPC_ *exoS*,* bla*_TEM_, *bla*_SHV_ *exo*S, *phz* M, *bla*_TEM_ *exo*S, *bla*_TEM_, *bla*_OXA−48_ *tox*A, *exo*S, *bla*_TEM_ *opr*L, *tox*A, *exo*S, *bla*_TEM_ *exo*S, *phz*M, *bla*_TEM_, *bla*_OXA−48_ *opr*L, *tox*A, *exo*S, *phz*M, *bla*_TEM_, *bla*_VIM_
Group 4	1 1 2 4 4 1 1 1 1	*opr*L, *tox*A, *exo*S, *bla*_TEM_ *opr*L, *tox*A, *exo*S, *phz*M, *bla*_TEM_ *opr*L, *tox*A, *exo* S, *phz*M, *bla*_TEM_ *opr*L, *tox*A, *exo*S, *phz*M, *bla*_TEM_, *bla*_CTX−M_ *opr*L, *tox*A, *exo*S, *phz*M, *bla*_TEM_,, *bla*_CTX−M_, *bla*_OXA−48_ *opr*L, *tox*A, *exo*S, *phz*M, *bla*_TEM_, *bla*_CTX−M_, *bla*_VIM_ *opr*L, *tox*A, *exo*S, *phz*M, *bla*_TEM_, *bla*_CTX−M_, *bla*_VIM,_*bla*_OXA−48_ *tox*A, *exo*S, *phz*M, *bla*_TEM_, *bla*_CTX−M_, *bla*_VIM_ *tox*A, *exo*S, *phz*M, *bla*_TEM_, *bla*_CTX−M_, *bla*_SHV_
Group 5	1 1 2 1 1 2 1 1 1 1 1	*exo*S *bla*_OXA−48_ *bla*_TEM_ *exo*S, *bla*_TEM_ *exo*S, *bla*_TEM_, *bla*_NDM_ *exo*S, *bla*_TEM_, *bla*_VIM_ *exo*S, *bla*_TEM_, *bla*_OXA−48_ *exo*S, *phz* M, *bla*_TEM_ *exo*S, *bla*_TEM_, *bla*_SHV_ *tox*A, *exo*S, *bla*_TEM_, *bla*_SHV_ *exo*S, *phz*M, *bla*_TEM_, *bla*_OXA−48_

### Phylogenetic Analysis of *P. aeruginosa opr*L Gene from Fish

**Fig. 4:**
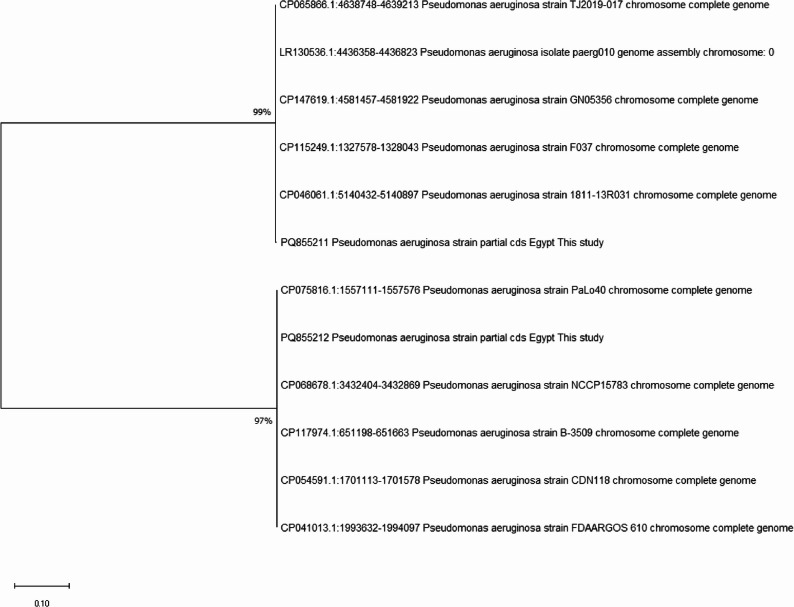
Phylogenetic relationships of *P. aeruginosa* isolates based on *opr*L gene sequences. The tree was constructed using the Maximum Likelihood method with 1000 bootstrap replicates. Fish-derived isolates clustered closely with human clinical strains, including both local (Egyptian) and international sequences retrieved from GenBank

Phylogenetic analysis of the *opr*L gene revealed close clustering of fish isolates with local and international human clinical strains, which may reflect potential genetic relatedness and zoonotic risk (Fig. 4).

## Discussion

*Pseudomonas aeruginosa* causes septicemia in freshwater fish and substantial aquaculture losses [21], and it poses zoonotic risks through exposure to contaminated water or fish products, particularly in immunocompromised individuals [17]. Climate-related environmental changes may further elevate these risks by enhancing bacterial survival and virulence, reinforcing the need for One Health monitoring [46]. In this study, its detection in 95.12% of fish samples reflects broad environmental circulation, consistent with Benie et al. [47], who reported a 91.1% prevalence in animal and fish products, and higher than the rates observed by Eissa et al. [48] and Mamdouh et al. [49]. Such variability may arise from differences in environmental conditions, sampling season, water quality, and hygiene management across farms [6, 17, 50]. These findings underscore the persistence and adaptability of *P. aeruginosa* in aquatic environments and highlight the need for improved biosecurity measures in aquaculture systems. From a One Health perspective, the widespread occurrence of this pathogen in aquaculture environments emphasizes the interconnected risks between aquatic ecosystems, animal health, and human exposure through direct contact or consumption of contaminated fish products [51].

Although this study found no significant correlation between *P. aeruginosa* occurrence and water temperature or pH, the bacterium’s adaptability allows growth across wide environmental ranges—temperatures up to 42 °C and pH 4.5–9.0 [14, 52]. However, 100% occurrence was recorded at 35 and 37 °C and pH 5.5 and 6.5, suggesting that suboptimal water conditions and elevated temperatures enhance bacterial persistence and proliferation. The relatively low pH value recorded in summer (5.5) may be related to seasonal accumulation of organic matter and increased microbial decomposition in aquaculture ponds [53]. Such conditions can lead to temporary acidification of pond water and act as an additional environmental stressor for Nile tilapia, potentially influencing host susceptibility and bacterial detectability [54]. Similar patterns were described by Doménech-Sánchez et al. [55] and other studies linking poor water quality and summer-associated temperature rises to increased pathogen prevalence [33, 52, 56]. These results suggest that management-related stressors, including shallow ponds and inadequate water exchange, may aggravate infection risk during warmer months [57, 58].

Environmental stressors not only influence bacterial abundance but may also affect the distribution of virulence-associated genes. The observed seasonal increase in virulent *P. aeruginosa* strains, particularly during early summer, suggests that temperature fluctuations may influence the prevalence of virulence determinants. The *exo*S gene was the most prevalent, consistent with previous reports [12, 30]. The *opr*L gene, essential for membrane integrity [59], showed higher detection at elevated temperatures, rising sharply at 39 °C. Similarly, *tox*A and *exo*S reached full prevalence at higher temperatures, indicating a greater presence of toxin-associated genes [60, 61]. The *phz*M gene, associated with phenazine toxin synthesis, also peaked during warmer conditions, which may support bacterial persistence [21]. These findings suggest that elevated temperatures may favor the presence of virulence determinants, potentially contributing to increased disease risk and zoonotic concerns [15, 62, 63].

Alongside increased virulence, rising temperatures from seasonal variation were associated with higher detection of antimicrobial resistance (AMR) genes in aquatic environments, raising public health concerns [64–66]. *Pseudomonas aeruginosa* can resist treatment via efflux pumps, β-lactamase and carbapenemase production, porin changes, target mutations, and biofilm formation [67], with ARG spread further promoted by microbial shifts, antibiotic degradation, and misuse [10, 68]. This study observed higher prevalence of ESBL genes *bla*_TEM_, *bla*_CTX-M,_ and *bla*_SHV_ during warmer periods, aligning with previous reports [21, 69], In contrast, *bla*_OXA-1_ was not detected in any season, which may reflect its predominance in clinical isolates rather than environmental strains [70, 71]. However, other OXA-type carbapenemases such as *bla*_OXA-48_ were detected, indicating that certain resistance determinants may circulate in aquaculture-associated *P. aeruginosa* even when others remain largely confined to clinical settings [72].

Carbapenemase genes, including *bla*_OXA−48_ and *bla*_VIM_ were detected more frequently during warmer sampling periods, while *bla*_NDM_ appeared seasonally, peaking in spring. The rare detection of *bla*_KPC_ suggests limited introduction from clinical environments and low horizontal transfer efficiency in local aquaculture [73–77]. These observations highlight the presence of ARGs in aquaculture systems and their potential for transmission through the food chain, reinforcing the relevance to global One Health concerns [78].

The co-occurrence of virulence and resistance determinants was most pronounced in isolates collected during the summer, when high temperatures and low pH coincided. Most isolates from this period harbored all four virulence genes alongside one or more β-lactamase or carbapenemase genes, indicating the frequent coexistence of virulence and resistance determinants under these environmental conditions [79, 80]. Such co-occurrence has been reported in *P. aeruginosa* and may contribute to bacterial persistence and adaptability in aquatic environments [81, 82]. These observations highlight the potential emergence of multidrug-resistant and virulent phenotypes in aquaculture systems and reinforce concerns regarding environmental and public health risks [83].

The phylogenetic relatedness between fish-derived and human-associated *P. aeruginosa* isolates observed in this study may suggest possible cross-species transmission and the zoonotic potential of aquaculture-derived strains. Similar patterns have been reported in other aquatic bacteria, such as *Aeromonas hydrophila*, which combine virulence and AMR traits [84]. Such convergence highlights the ecological resilience of aquatic pathogens and the potential role of aquaculture systems in harboring clinically relevant resistance genes [85].

Overall, this study highlights the complex interplay between environmental conditions, virulence, and antimicrobial resistance in *P. aeruginosa* within aquaculture systems. Fish samples were analyzed in pooled composites to enhance molecular screening efficiency, which may limit resolution and the precise estimation of individual-level prevalence. Seasonal temperature and pH fluctuations appeared to influence the detection of virulence and resistance genes, with potential implications for fish health and public safety. However, these findings should be interpreted cautiously due to the limited sample size and geographic scope.

These results underscore the importance of implementing effective aquaculture management practices and prudent antimicrobial use to mitigate the emergence and spread of resistant and virulent strains. Targeted interventions, including strengthened biosecurity, exploration of alternative therapies, and vaccination strategies, may further reduce pathogen prevalence and support sustainable aquaculture. Future research should incorporate larger-scale longitudinal studies using advanced molecular tools such as qPCR, metagenomics, and whole-genome sequencing. Integrating phenotypic and genotypic approaches, together with expanded phylogenetic analyses including a broader set of isolates, would help to better elucidate the drivers of AMR and virulence evolution in aquaculture ecosystems. These recommendations align with the One Health framework, linking environmental monitoring, animal health, and public safety [86, 87].

## Conclusion

Seasonal environmental changes, particularly higher temperatures and lower pH, appear to be associated with the co-occurrence of virulence and resistance genes in *P. aeruginosa* from Egyptian aquaculture. These results emphasize the importance of integrated surveillance and management strategies to mitigate pathogen emergence and antimicrobial resistance risks. Future studies should incorporate larger, multi-site, longitudinal monitoring combined with advanced molecular diagnostics to better understand the dynamics of these pathogens in aquaculture systems.

## Data Availability

All the data generated or analyzed in this study are included in this published article.The sequences of the *opr* L gene were deposited in the National Center for Biotechnology Information (NCBI) GenBank database under accession numbers PQ855211 and PQ855212.

## References

[1] Bunting SW. Principles of sustainable aquaculture: promoting social, economic and environmental resilience. Routledge; 2013.

[2] O’Shea T, Jones R, Markham A, Norell E, Scott J, Theuerkauf S, Waters T. Towards a Blue Revolution: Catalyzing Private Investment in Sustainable Aquaculture Production Systems. Virginia: The Nature Conservancy and Encourage Capital, Arlington; 2019.

[3] Mallick A, Panigrahi AK. Effect of temperature variation on disease proliferation of common fishes in perspective of climate change. Int J Exp Res Rev. 2018;16:40–9.

[4] Sarà G, Gouhier TC, Brigolin D, Porporato EM, Mangano MC, Mirto S, Pastres R. Predicting shifting sustainability trade-offs in marine finfish aquaculture under climate change. Glob Change Biol. 2018;24(8):3654–65. 10.1111/gcb.14296 29723929

[5] Maulu S, Hasimuna OJ, Haambiya LH, Monde C, Musuka CG, Makorwa TH, Nsekanabo JD. Climate change effects on aquaculture production: sustainability implications, mitigation, and adaptations. Front Sustainable Food Syst. 2021;5:609097.

[6] Cascarano MC, Stavrakidis-Zachou O, Mladineo I, Thompson KD, Papandroulakis N, Katharios P. Mediterranean aquaculture in a changing climate: Temperature effects on pathogens and diseases of three farmed fish species. Pathogens. 2021;10(9):1205. 34578236 10.3390/pathogens10091205 PMC8466566

[7] Gao K, Gao G, Wang Y, Dupont S. Impacts of ocean acidification under multiple stressors on typical organisms and ecological processes. Marine Life Science & Technology; 2020:2;279–91.

[8] Okon EM, Oyesiji AA, Okeleye DE, Kanonuhwa M, Khalifa NE, Eissa ESH, Abdelnour SA. The Escalating threat of climate change-driven diseases in fish: Evidence from a global perspective–A literature review. Environ Res, 2024, 120184. 10.1016/j.envres.2024.120184 39426450

[9] Ficke AD, Myrick CA, Hansen LJ. Potential impacts of global climate change on freshwater fisheries. Rev Fish Biol Fisheries. 2007;17:581–613.

[10] Pepi M, Focardi S. Antibiotic-resistant bacteria in aquaculture and climate change: A challenge for health in the Mediterranean area. Int J Environ Res Public Health. 2021;18(11):5723. 34073520 10.3390/ijerph18115723 PMC8198758

[11] Hanna MI, El-Hady MA, Hanaa AA, Elmeadawy SA, Kenwy AM. Contribution on Pseudomonas aeruginosa infection in African Catfish (Clariasgariepinus). Res J Pharm Biol Chem Sci. 2014;5(5):575–88.

[12] Tawab AE, Maarouf AA, A. A., Ahmed NM. Detection of Virulence factors of *Pseudomonas species* isolated from fresh water fish by PCR. Benha Veterinary Med J. 2016;30(1):199–207.

[13] Elshafiee EA, Khalefa HS, Al-Atfeehy NM, Amer F, Hamza DA, Ahmed ZS. Biofilms and efflux pump regulatory gene (mexR) in multidrug-resistant *Pseudomonas aeruginosa* isolated from migratory birds in Egypt. Veterinary World. 2022;15(10):2425. 36425141 10.14202/vetworld.2022.2425-2431 PMC9682394

[14] Tribelli PM, López NI. Insights into the temperature responses of *Pseudomonas* species in beneficial and pathogenic host interactions. Applied Microbiol Biotechnol. 2022;106(23):7699–709. 10.1007/s00253-022-12243-z 36271255

[15] Barbier M, Damron FH, Bielecki P, Suárez-Diez M, Puchałka J, Albertí S, Goldberg JB. From the environment to the host: re-wiring of the transcriptome of *Pseudomonas aeruginosa* from 22 C to 37 C. PLoS ONE, 2014, 9(2), e89941. 10.1371/journal.pone.0089941 PMC3933690 24587139

[16] Abdullahi IN, Mejri S, Okwume CC, Lawal NA, Olusegun OA, Sallem RB, Slama KB. Global epidemiology of high priority and pandemic Pseudomonas aeruginosa in pets, livestock, wild, and aquatic animals: a systematic review and meta-analysis. Lett Appl Microbiol. 2025;78(3):ovaf028. 39999856 10.1093/lambio/ovaf028

[17] Sheng L, Wang L. The microbial safety of fish and fish products: Recent advances in understanding its significance, contamination sources, and control strategies. Compr Rev Food Sci Food Saf. 2021;20(1):738–86. 33325100 10.1111/1541-4337.12671

[18] Qin S, Xiao W, Zhou C, Pu Q, Deng X, Lan L, Liang H, Song X, Wu M. *Pseudomonas aeruginosa*: pathogenesis, virulence factors, antibiotic resistance, interaction with host, technology advances and emerging therapeutics. Sig Transduct Target Ther. 2022;7:199. 10.1038/s41392-022-01056-1. 10.1038/s41392-022-01056-1 PMC9233671 35752612

[19] Michalska M, Wolf P. *Pseudomonas* Exotoxin A: optimized by evolution for effective killing. Front Microbiol. 2015;15:6:963. https://doi.org/10.3389/fmicb.2015.00963 . PMID: 26441897; PMCID: PMC4584936. 10.3389/fmicb.2015.00963 PMC4584936 26441897

[20] Rao L, De La Rosa I, Xu Y, Sha Y, Bhattacharya A, Holtzman MJ. … Eissa, N. T. *Pseudomonas aeruginosa* survives in epithelia by ExoS-mediated inhibition of autophagy and mTOR. EMBO Rep, 2021, 22(2), e50613. 10.15252/embr.202050613 PMC7857434 33345425

[21] Algammal AM, Mabrok M, Sivaramasamy E, Youssef FM, Atwa MH, El-Kholy AW, Hozzein WN. Emerging MDR-*Pseudomonas aeruginosa* in fish commonly harbor opr L and tox A virulence genes and *bla*_TEM_ and *bla*_CTX-M_, and *tet* A antibiotic-resistance genes. Sci Rep. 2020;10(1):15961. 32994450 10.1038/s41598-020-72264-4 PMC7524749

[22] Sahoo SR, Pradhan AK, Das RP, Panigrahi LL, Arakha M. Pyocyanin is the microbial blue-green pigment: a review on its history, virulence, and therapeutic use. Curr Bioact Compd. 2023;19(6):62–76.

[23] Nikbin V, et al. Molecular identification and detection of virulence genes among *Pseudomonas aeruginosa* isolated from different infectious origins. Iran J Microbiol. 2012;4:118. 23066485 PMC3465536

[24] Khan F. Multifaceted strategies for alleviating *Pseudomonas aeruginosa* infection by targeting protease activity: Natural and synthetic molecules. Int J Biol Macromol, 2024, 134533. 10.1016/j.ijbiomac.2024.134533 39116989

[25] Grudniak AM, Klecha B, Wolska KI. Effects of null mutation of the heat-shock gene htpG on the production of virulence factors by *Pseudomonas aeruginosa*. Future Microbiol. 2018;13(1):69–80. 29199454 10.2217/fmb-2017-0111

[26] Marouf S, Li X, Salem HM, Ahmed ZS, Nader SM, Shaalan M, Cheang T. Molecular detection of multidrug-resistant *Pseudomonas aeruginosa* of different avian sources with pathogenicity testing and in vitro evaluation of antibacterial efficacy of silver nanoparticles against multidrug-resistant *P. aeruginosa*. Poult Sci. 2023;102(10):102995. 37566970 10.1016/j.psj.2023.102995 PMC10440575

[27] Shahrokhi GR, Rahimi E, Shakerian A. The prevalence rate, pattern of antibiotic resistance, and frequency of virulence factors of *Pseudomonas aeruginosa* strains isolated from fish in Iran. J Food Qual, 2022(1), 8990912.

[28] Schar D, Klein EY, Laxminarayan R, Gilbert M, Van Boeckel TP. Global trends in antimicrobial use in aquaculture. Sci Rep. 2020;10(1):1–9. 33318576 10.1038/s41598-020-78849-3 PMC7736322

[29] Hamza D, Zaher HM. Carriage of Rifampicin-and Multidrug-Resistant *Pseudomonas aeruginosa* in Apparently Healthy Camels: A View Through a Zoonosis Lens. Microbiol Res. 2025;16(6):107.

[30] Suresh K, Pillai D, Soman M, Sreenivas A, Paul R. Isolation and identification of antimicrobial susceptibility, biofilm formation, efflux pump activity, and virulence determinants in multi-drug resistant *Pseudomonas aeruginosa* isolated from freshwater fishes. J Water Health. 2023;21(12):1858–70. 38153717 10.2166/wh.2023.206

[31] Elshafiee EA, Nader SM, Dorgham SM, Hamza DA. Carbapenem-resistant originating from farm animals and people in Egypt. J veterinary Res. 2019;63(3):333–7. 10.2478/jvetres-2019-0049 PMC6749737 31572812

[32] Hammoudi Halat D, Ayoub Moubareck C. The current burden of carbapenemases: review of significant properties and dissemination among gram-negative bacteria. Antibiotics. 2020;9(4):186. 32316342 10.3390/antibiotics9040186 PMC7235769

[33] Ayoub HF, Khafagy AR, Esawy AM, El-Moaty NA, Alwutayd KM, Mansour AT, El-Tarabili RM. Phenotypic, molecular detection, and Antibiotic Resistance Profile (MDR and XDR) of *Aeromonas hydrophila* isolated from Farmed Tilapia zillii and Mugil cephalus. BMC Vet Res. 2024;20(1):84. 38459543 10.1186/s12917-024-03942-y PMC10921648

[34] Masson-Delmotte V, Zhai P, Pörtner HO, Roberts D, Skea J, Shukla PR, Waterfield T. Global warming of 1.5 C. An IPCC Special Report on the impacts of global warming of, 2019, 1, 93–174.3.

[35] Burnham JP. Climate change and antibiotic resistance: a deadly combination. Therapeutic Adv Infect Disease. 2021;8:2049936121991374. 10.1177/2049936121991374 PMC7890742 33643652

[36] Hossain A, Habibullah-Al-Mamun M, Nagano I, Masunaga S, Kitazawa D, Matsuda H. Antibiotics, antibiotic-resistant bacteria, and resistance genes in aquaculture: risks, current concern, and future thinking. Environmental Science and Pollution Research; 2022. pp. 1–22. 10.1007/s11356-021-17825-4 35028843

[37] Tang Y, Stratton CW. Advanced Techniques in Diagnostic Microbiology. Springer Science and Business Media, LLC.Printed in the United States of America. Volume 9. TB/EB); 2006. p. 7.

[38] Gautam T, Isolation. Speciation and Detection of Virulence Factors *in Pseudomonas* Species with Special Reference to Metallo-Betalactamase Production (Doctoral dissertation, Rajiv Gandhi University of Health Sciences (India)). 2019.

[39] Oliveira CF, Paim TG, Reiter KC, Rieger A, D’Azevedo PA. Evaluation of four different DNA extraction methods in coagulase-negative staphylococci clinical isolates. Rev Inst Med Trop Sao Paulo Jan-Feb. 2014;56(1):29–33. PMID: 24553605; PMCID: PMC4085835. 10.1590/S0036-46652014000100004 PMC4085835 24553605

[40] Hassan KI, Abdullah SR. Detection of Pseudomonas aeruginosa in Clinical Samples Using PCR Targeting ETA and gyrB Genes. Baghdad Sci J. 2018;15(4):Article5.

[41] Monstein HJ, Östholm-Balkhed Å, Nilsson MV, Nilsson M, Dornbusch K, Nilsson LE et al. Multiplex PCR amplification assay for the detection of *bla*_SHV_, *bla*_TEM_, and *bla*_CTX-M_ genes in *Enterobacteriaceae.* Apmis. 2007, 115: 1400–1408. 10.1111/j.1600-0463.2007.00722.x 10.1111/j.1600-0463.2007.00722.x 18184411

[42] Djeffal S, Bakour S, Mamache B, Elgroud R, Agabou A, Chabou S, Hireche S, Bouaziz O, Rahal K, Rolain JM, et al. Prevalence and clonal relationship of ESBL-producing Salmonella strains from humans and poultry in northeastern Algeria. BMC Vet Res. 2017;13:1–9. 10.1186/s12917-017-1050-3. 28506272 10.1186/s12917-017-1050-3 PMC5433073

[43] Li B, Yi Y, Wang Q, Woo PC, Tan L, Jing H, Gao GF, Liu CH, et al., et al. Analysis of drug resistance determinants in Klebsiella pneumoniae isolates from a tertiary-care hospital in Beijing, China. PLoS ONE. 2012(a);7:e42280. 10.1371/journal.pone.0042280. 10.1371/journal.pone.0042280 PMC3409176 22860106

[44] Li J, Hu Z, Hu Q et al. Isolation of the first IMP-4 metallo-β-lactamase producing *Klebsiella pneumoniae* in Tianjin, China. Braz. J. Microbiol. 2012(b); 43: 917–922. 10.1590/S1517-83822012000300010 10.1590/S1517-838220120003000010 PMC3768900 24031907

[45] Dallenne C, Da Costa A, Decré D, Favier C, Arlet G, et al. Development of a set of multiplex PCR assays for the detection of genes encoding important β-lactamases in *Enterobacteriaceae*. J Antimicrob Chemother. 2010;65:490–5. 10.1093/jac/dkp498. 20071363 10.1093/jac/dkp498

[46] Singh S, Sharma P, Pal N, Sarma DK, Tiwari R, Kumar M. Holistic one health surveillance framework: synergizing environmental, animal, and human determinants for enhanced infectious disease management. ACS Infect Dis. 2024;10(3):808–26. 38415654 10.1021/acsinfecdis.3c00625

[47] Benie CKD, Dadié A, Guessennd N, Kouadio NA, Kouame ND, N’golo DC, Aka S, Dako E, Dje KM, Dosso M. Characterization of virulence potential of *Pseudomonas aeruginosa* isolated from bovine meat, fresh fish, and smoked fish. Eur J Microbiol Immunol 2017, 7 (1): 55–64. 10.1556/1886.2016.00039 PMC5372481 28386471

[48] Eissa NME, Abou EEN, Shaheen AA, Abbass A. Characterization of *Pseudomonas Species* Isolated from Tilapia *Oreochromis niloticus* in Qaroun and Wadi-El-Rayan Lakes, Egypt. Global Vet. 2010;5:116–21.

[49] Mamdouh D, Hassan MA, Fawzy EE. Bacterial evaluation of the quality of farmed fish in Kafr El- Sheikh City in Egypt.Benha Vet. Med J. 2022;41:16–21.

[50] Bedane TD, Agga GE, Gutema FD. Hygienic assessment of fish handling practices along production and supply chain and its public health implications in Central Oromia. Ethiopia Sci Rep. 2022;12(1):13910. 35977962 10.1038/s41598-022-17671-5 PMC9385613

[51] Omonofa AS, Kayode-Edwards II, Isibor PO. Introduction to Freshwater Ecosystems and the One Health Framework. In Pollution Tolerance of Freshwater Ecosystems and Biomonitoring, 2026 (pp. 1–17). CRC.

[52] Mozaheb N, Rasouli P, Kaur M, Van Der Smissen P, Larrouy-Maumus G, Mingeot-Leclercq M. P. A mildly acidic environment alters *Pseudomonas aeruginosa* virulence and causes remodeling of the bacterial surface. Microbiol Spectr. 2023;11(4):e04832–22. 37278652 10.1128/spectrum.04832-22 PMC10433952

[53] Hu M, Sardans J, Le Y, Yan R, Peñuelas J. Coastal wetland conversion to aquaculture pond reduced soil P availability by altering P fractions, phosphatase activity, and associated microbial properties. Chemosphere. 2023;311:137083. 36334732 10.1016/j.chemosphere.2022.137083

[54] Abd El-Hack ME, El-Saadony MT, Nader MM, Salem HM, El-Tahan AM, Soliman SM, Khafaga AF. Effect of environmental factors on growth performance of Nile tilapia (*Oreochromis niloticus*). Int J Biometeorol. 2022;66(11):2183–94. 36044083 10.1007/s00484-022-02347-6 PMC9640449

[55] Doménech-Sánchez A, Laso E, Albertí S. Prevalence and Control of *Pseudomonas aeruginosa* in Tourist Facilities across the Canary Islands, Spain. Pathogens. 2024;13(6):501. 38921799 10.3390/pathogens13060501 PMC11207077

[56] Thorstad, E. B., Bliss, D., Breau, C., Damon-Randall, K., Sundt‐Hansen, L. E., Hatfield,E. M., … Sutton, S. G. Atlantic salmon in a rapidly changing environment—Facing the challenges of reduced marine survival and climate change. Aquatic Conservation: Marine and Freshwater Ecosystems, 2021; 31(9), 2654–2665.

[57] Can, E., Austin, B., Steinberg, C., Carboni, C., Sağlam, N., Thompson, K., … Ergün,S. E. B. A. H. A. T. T. İ. N. Best practices for fish biosecurity, well-being and sustainable aquaculture. Sustainable Aquatic Research, 2023, 2(3).

[58] Mramba RP, Kahindi EJ. Pond water quality and its relation to fish yield and disease occurrence in small-scale aquaculture in arid areas. Heliyon, 2023, 9(6). 10.1016/j.heliyon.2023.e16753 PMC10238929 37274696

[59] AbdEl-Maogoud H, Edris AM, Mahmoud AH, Maky MA. Occurrence and characterization of *Pseudomonas species* isolated from Fish Marketed in Sohag Governorate, Egypt.SVU- Inter. J Vet Sci. 2021;4(2):76–84.

[60] Fadhil L, Al-Marzoqi AH, Zahraa MA, Shalan AA. Molecular and phenotypic study of virulence genes in a pathogenic strain of *Pseudomonas aeruginosa* isolated from various clinical origins by PCR: profiles of genes and toxins. Res J Pharm Biol Chem Sci. 2016;7:590–8.

[61] Aljebory IS. PCR detection of some virulence genes of *Pseudomonas aeruginosa* in Kirkuk city. Iraq J Pharm Sci Res. 2018;10:1068–71.

[62] Maimona S, Sabiel YA. De-tection of the Causative Agents of Bacterial Fish Septicemia of Tilapia and Clarais in Khartoum State. International Journal of Re-cent Scientific Research Vol. 6, 2015; Issue, 6, pp.4374–4377.

[63] Adel M, Rashed MA. Detection of some virulence genes of *Pseudomonas* spp. in some apparently healthy cultured Nile Tilapia in Kafr EL Sheikh Governorate.

[64] Rodríguez-Verdugo A, Lozano-Huntelman N, Cruz-Loya M, Savage V, Yeh P. Compounding effects of climate warming and antibiotic resistance. IScience, 2020; 23(4). 10.1016/j.isci.2020.101024 PMC7160571 32299057

[65] Mukhopadhyay S, Karmakar R, Chakrabarti S, Ghosh MM, Ganguli S. Evaluating the Impact of Climate Change on Antimicrobial Resistance and Rise in Dysentery Using Next Generation Sequencing Based Approaches. In Microbiology-2.0 Update for a Sustainable Future 2024 (pp. 373–93). Singapore: Springer Nature Singapore.

[66] World Health Organization. Global Antibiotic Resistance Surveillance Report 2025. Geneva: World Health Organization; 2025.

[67] Fernández-Billón M, Llambías-Cabot AE, Jordana-Lluch E, Oliver A, Macià MD. Mechanisms of antibiotic resistance in *Pseudomonas aeruginosa* biofilms. Biofilm. 2023;5:100129. 37205903 10.1016/j.bioflm.2023.100129 PMC10189392

[68] FAO. Antimicrobials: Handle with Care. FAO Publication. Rome: FAO; 2025.

[69] Mohamed DS, Ragab AM, Ibrahim MS, Talat D. Prevalence and Antibiogram of *Pseudomonas aeruginosa* Among Nile Tilapia and Smoked Herring, with an Emphasis on their Antibiotic Resistance Genes (*bla*_TEM_, *bla*_SHV_, *bla*_OXA-1_ and *amp*C) and Virulence Determinant (*opr*L and *tox*A). J Adv Veterinary Res. 2023;13(6):1166–72.

[70] Ishida Y, Ahmed AM, Mahfouz NB, Kimura T, El-Khodery SA, Moawad AA, Shimamoto T. Molecular analysis of antimicrobial resistance in Gram-negative bacteria isolated from fish farms in Egypt. J Vet Med Sci. 2010;72(6):727–34. 20145377 10.1292/jvms.09-0538

[71] Rahman A. Molecular profiling, antibiotic susceptibility patterns and pathogenic traits of ESBL producing *Acinetobacter baumannii* Isolated from wastewater discharges from Goranchatbari sub-catchment area in Dhaka city (Doctoral dissertation, Brac University), 2024.

[72] Lu Y, Zhang B, Wang Z, He Y, Ge H, Ma H, Cui P. From Host-Derived Pressures to the Environmental Anti-Antimicrobial Peptides Resistome: Mechanisms, Reservoirs and Implications for Therapeutic Peptide Design. Mar Drugs. 2026;24(2):76. 41745479 10.3390/md24020076 PMC12942635

[73] Ramírez-Castillo FY, Guerrero-Barrera AL, Avelar-González FJ. An overview of carbapenem-resistant organisms from food-producing animals, seafood, aquaculture, companion animals, and wildlife. Front Veterinary Sci. 2023;10:1158588. 10.3389/fvets.2023.1158588 PMC10311504 37397005

[74] Mohamed S, Alobied A, Hussien W, Saeed M. *bla*_OXA-48_ carbapenem resistant *Pseudomonas aeruginosa* clinical isolates in Sudan. J Adv Microbiol. 2018;10(4):1–5.

[75] Dewi DAR, Götz B, Thomas T. Diversity and genetic basis for carbapenem resistance in a coastal marine environment. Appl Environ Microbiol. 2020;86(10):e02939–19. 32198174 10.1128/AEM.02939-19 PMC7205498

[76] Jung H, Pitout JD, Matsumura Y, Strydom KA, Kingsburgh C, Ehlers MM, Kock MM. Genomic epidemiology and molecular characteristics of *bla*_NDM_-1-positive carbapenem-resistant *Pseudomonas aeruginosa* belonging to international high-risk clone ST773 in the Gauteng region, South Africa. Eur J Clin Microbiol Infect Dis. 2024;43(4):627–40. 38265603 10.1007/s10096-024-04763-5 PMC10965571

[77] Luca L. Detection of carbapenem-resistant *Enterobacterales* in food producing animals and human patients. A One Health perspective; 2024.

[78] Rubin JE, Ekanayake S, Fernando C. Carbapenemase-producing organism in food. Emerg Infect Dis. 2014;20(7):1264. 24960459 10.3201/eid2007.140534 PMC4073846

[79] Kaba HE, Kuhlmann E, Scheithauer S. Thinking outside the box: Association of antimicrobial resistance with climate warming in Europe–A 30 country observational study. Int J Hyg Environ Health. 2020;223(1):151–8. 31648934 10.1016/j.ijheh.2019.09.008

[80] Ahmed, F., Mirani, Z. A., Mirani, P. N., Imdad, M. J., Khan, F. Z., Khan, M. N., …Zhao, Y. Pseudomonas aeruginosa response to acidic stress and imipenem resistance. Applied Sciences, 2022, 12(16),8357.

[81] Méndez-Cea, B., García-García, I., Gazol, A., Camarero, J. J., de Andrés, E. G., Colangelo,M., … Linares, J. C. Weak genetic differentiation but strong climate-induced selective pressure toward the rear edge of mountain pine in north-eastern Spain. Science of the Total Environment, 2023, 858, 159778. 10.1016/j.scitotenv.2022.159778 36309267

[82] de Pereira O. T. Influence of Environmental Cues into the Quorum Sensing Regulatory Network in the Opportunistic Pathogen *Pseudomonas aeruginosa* (Doctoral dissertation, Université du Québec, Institut national de la recherche scientifique), 2024.

[83] Zambrano MM. Interplay between antimicrobial resistance and global environmental change. Annu Rev Genet. 2023;57(1):275–96. 37708420 10.1146/annurev-genet-022123-113904

[84] Nhinh DT, Le DV, Van KV, Huong Giang NT, Dang LT, Hoai TD. Prevalence, virulence gene distribution and alarming the multidrug resistance of Aeromonas hydrophila associated with disease outbreaks in freshwater aquaculture. Antibiotics. 2021;10(5):532. 34064504 10.3390/antibiotics10050532 PMC8147934

[85] Deng Y, Tan A, Zhao F, Wang F, Gong H, Lai Y, Huang Z. Global distribution of antimicrobial resistance genes in aquaculture. One Health Adv. 2025;3(1):6.

[86] MacFadden DR, McGough SF, Fisman D, Santillana M, Brownstein JS. Antibiotic resistance increases with local temperature. Nat Clim Change. 2018;8(6):510–4. 10.1038/s41558-018-0161-6 PMC6201249 30369964

[87] Larsson DG, Flach C. F.Antibiotic resistance in the environment. Nature Reviews Microbiol. 2022;20(5):257–69. 10.1038/s41579-021-00649-x PMC8567979 34737424

